# Choose your side in orthognathic surgery: YouTube™ or TikTok™

**DOI:** 10.1186/s12903-026-08071-6

**Published:** 2026-03-09

**Authors:** Aylin Pasaoglu Bozkurt, Mehmet Kiziloglu, Abdullah Enes Ilgun

**Affiliations:** 1 Department of Orthodontics, Faculty of Dentistry, Istanbul Aydin University, Istanbul, 34295 Turkey; 2 DDS, Private Practice, Kayseri, Turkey

**Keywords:** Orthognathic Surgery, TikTok, YouTube, Orthodontics, Dentistry

## Abstract

**Aim:**

This study aims to assess the reliability and overall quality of publicly accessible video content related to orthognathic surgery on two major social media platforms: TikTok™ and YouTube™.

**Method:**

A total of 320 videos were systematically analyzed on TikTok™ and YouTube™ using the search terms “jaw surgery” and “orthognathic surgery.” Data collection was carried out between March and April 2024. Each video was evaluated using the Global Quality Score (GQS) to determine general content quality and a modified version of the DISCERN instrument to assess reliability. Video reliability was assessed using the DISCERN instrument, a standardized tool for evaluating the quality of health information. Additional parameters, including video duration, view count, number of likes, comments, and uploader type were recorded and analyzed. All searches were performed in incognito mode without VPN to minimize algorithmic bias and ensure reproducibility.

**Results:**

For the ‘jaw surgery’ term, statistically significant associations were observed between uploader type and platform (p < 0.05): patients were the predominant uploaders on TikTok, whereas doctors and clinics were more common on YouTube. Comparative analyses demonstrated that GQS and DISCERN scores were comparable between platforms, with TikTok showing slightly higher mean scores for the ‘Orthognathic Surgery’ term, though these differences were not statistically significant (*p* > 0.05). YouTube videos were longer in duration for certain comparisons, whereas TikTok videos received significantly higher engagement metrics (views, likes, comments, and subscriber counts) (*p* < 0.05).

**Conclusion:**

The content about orthognathic surgery on both platforms was found to be of poor to moderate quality (mean GQS: 1.40–2.49 out of 5) and limited reliability (mean DISCERN: 0.81–2.21 out of 4), with significant variation between search terms and platforms.

**Clinical trial number:**

This study did not involve human participants, identifiable personal data, or experimental interventions. All analyzed material consisted of publicly accessible online videos, which are exempt from institutional review board approval according to relevant guideline (e.g., the Declaration of Helsinki and local regulations). Therefore, ethical approval was not required and was not sought.

## Introduction

Orthognathic surgery is a surgical intervention designed to treat skeletal discrepancies, dental malocclusions, and facial aesthetic concerns. These procedures aim to improve both functional outcomes (such as mastication, speech, respiration) and facial harmony. Commonly performed procedures include Le Fort I osteotomy, bilateral sagittal split osteotomy, and genioplasty. The postoperative period typically involves a soft diet, strict oral hygiene, and regular follow-up, with full recovery often requiring several months [1]. Although transient complications such as swelling, pain, and temporary paresthesia are common, the long-term outcomes are often reported as favorable, with many patients describing improvements in oral functions and quality of life [2].

Given the complexity of orthognathic surgery and the lengthy recovery period, accurate and accessible patient education is essential. Social media platforms have emerged as useful tools for sharing medical experiences and information. Content generated by healthcare professionals, patients, or general public can inform and support individuals preparing for or recovering from surgery. Such content may help establish realistic expectations, reduce anxiety, and enhance patient engagement in the treatment process [3, 4].

In addition to peer-reviewed literature, an increasing number of educational resources are available through visual and interactive formats. Medical videos, surgical demonstrations, and patient testimonials are widely circulated on social media, particularly on platforms such as TikTok™ and YouTube™, where video content dominates [5–7]. These platforms enable rapid dissemination of health-related information ; however, they also pose considerable challenges to the accuracy, credibility, and ethical integrity of the presented content [8–10].

TikTok™ and YouTube™ differ substantially in terms of video formats, user demographics, and engagement dynamics. YouTube, established in 2005, is primarily oriented toward long-form videos, with an audience that spans a wide range of age groups and is often seeking structured, in-depth content. In contrast, TikTok, launched globally in 2018, specializes in short-form videos optimized for rapid consumption and algorithm-driven discoverability, with a user base dominated by younger demographics, particularly those aged 16–34 [11–16]. These differences affect the way users engage with health-related information: while YouTube encourages prolonged viewing and discussion, TikTok’s brief, visually oriented videos tend to elicit higher rates of likes and shares [15–17]. Social media algorithms, designed to maximize user engagement, often prioritize content based on user preferences rather than informational value.

The increasing proliferation of medical information on social media introduces several ethical and professional challenges. Misinformation, especially when presented in a seemingly authoritative format, may lead to inappropriate self-diagnosis, treatment delays, or loss of trust in healthcare professionals. Thus, it is important that health information shared on digital platforms adheres to ethical standards and regulatory frameworks that prioritize patient safety and public health [18–21].

Although several studies have examined the reliability of health-related content on YouTube™ or TikTok™ individually [23, 26, 28, 29], there is still a lack of direct comparative analyses between the two platforms, particularly in the context of maxillofacial or orthodontic surgery. This study addresses this gap by systematically comparing the reliability, informative value, and engagement metrics of orthognathic surgery-related videos on both TikTok™ and YouTube™ using validated scoring systems (GQS and modified DISCERN).

This comparative design highlights how platform-specific features—such as video format, algorithmic personalization, and audience age—affect educational effectiveness and content credibility. Understanding these patterns is important for clinicians and educators seeking to guide patients toward reliable online sources.

In addition, this study underscores the importance of healthcare professionals’ active participation in social media to ensure evidence-based, ethically sound dissemination of surgical information. By analyzing how different video-sharing platforms represent orthognathic surgery, the findings may help clinicians develop strategies to balance public engagement with scientific accuracy in digital communication.

Therefore, the present study aims to evaluate the informational value and overall quality of orthognathic surgery-related videos on TikTok™ and YouTube™, two widely used video-based platforms. The results may guide future strategies to improve the accuracy, accessibility, and educational impact of online health information.

## Materials and methods

This study did not involve any human participants, identifiable personal data, or experimental interventions. All videos analyzed were publicly accessible on YouTube™ and TikTok™. According to institutional and national research ethics guidelines, research based solely on publicly available data does not require institutional review board (IRB) approval. Therefore, ethical approval was not required and was not obtained.

### Search strategy

Videos related to orthognathic surgery were identified using the search terms “jaw surgery” and “orthognathic surgery,” entered separately into TikTok™ and YouTube™. For each term, the first 80 videos meeting the inclusion criteria were selected, resulting in a total of 320 videos. To minimize algorithmic personalization bias, all searches were performed once in incognito mode without logging into any account, between March 1 and April 1, 2024. All searches were performed from Istanbul, Türkiye, without the use of a VPN, to ensure transparency and reproducibility of the process. Search results were archived immediately after collection to prevent future algorithm-driven changes. To confirm consistency, the same search procedure was repeated one week later under identical conditions, and no substantial differences were observed in the retrieved video lists.

### Inclusion and exclusion criteria

#### Videos were included if they

1. Were in English,
2. Contained educational or clinical information about orthognathic (jaw) surgery,
3. Had clear visual or verbal content.

Videos containing only background music were also included if their visuals conveyed relevant information.

Videos were excluded if they:

- Were duplicates or irrelevant to the topic,
- Contained only advertisements,
- Had no explanatory audio or meaningful visuals,
- Were repetitive or off-topic.

Two independent reviewers screened all videos, and any disagreements were resolved through discussion and consensus.

#### Scoring and evaluation tools

Each video was assessed using two validated instruments:

1. Global Quality Score (GQS) — evaluates the overall educational quality on a 5-point scale (1 = very poor; 5 = excellent).
2. Modified DISCERN Tool — measures the reliability of medical information based on four key criteria (0–4 points in total). (Table 1).

**Table 1:** Modified DISCERN Scoring Criteria

Criterion	Description	Score (0–1)
1. Source citation	Does the video clearly identify information sources such as references, guidelines, or experts?	1 = Yes; 0 = No
2. Objectivity	Is the content presented impartially without exaggerated or biased claims?	1 = Yes; 0 = No
3. Presentation of options	Does the video discuss alternative treatment options or approaches?	1 = Yes; 0 = No
4. Balance of risks and benefits	Are potential risks and benefits presented in a balanced and factual way?	1 = Yes; 0 = No

Each “Yes” answer received one point, yielding a total score from 0 (no criteria met) to 4 (all criteria met). Higher scores indicated greater reliability.

The GQS criteria were used to assess content quality and usefulness for patients. (Table 2).

**Table 2:** Global Quality Score (GQS) Criteria

Score	Description
1	Poor quality, poor flow, most information missing, not useful
2	Generally poor, limited use
3	Moderate quality, some information adequately discussed
4	Good quality, informative and useful
5	Excellent quality, highly useful and comprehensive

Both reviewers independently applied the GQS and DISCERN scoring tools.

The inter-rater reliability was calculated using Cohen’s kappa, yielding a value of 0.95, indicating excellent agreement.

#### Search filters

#### To maintain comparability across platforms

- On YouTube™, the “All time” and “Most viewed” filters were used.
- On TikTok™, the “All time” and “Most liked” filters were applied because the “Most viewed” option was unavailable.

Although these filters cannot be fully standardized, they provide the most consistent framework for evaluating cross-platform visibility and engagement.

No video duration filter was applied; both short- and long-form content were included. Videos were analyzed in the default ranking order of each platform without manual reordering.

### Content categorization

Videos were classified into six predefined thematic categories:

1. Case evaluation,
2. Experience (personal patient narratives),
3. Change (surgical transformation/before-after content),
4. Animation,
5. Surgery (surgical procedure footage),
6. Recommendation.

This categorization aimed to identify trends in communicative intent and distinguish patient-centered narratives from clinician-generated educational material.

### Statistical analysis

In this study, descriptive statistics were obtained. The assumption of normal distribution was assessed using the Shapiro–Wilk test. When the assumption of normality was not met, the Mann-Whitney U test was applied to compare two independent groups. In testing the relationship between categorical variables, the Pearson Chi-Square test was used when the sample size assumption (expected cell frequency > 5) was met, and the Fisher’s Exact test was used when the sample size assumption was not met. All statistical analyses were performed using IBM SPSS Statistics version 25. Significance level was taken as *p* < 0.05.

## Results

### Platform-wise comparisons

Statistically significant differences were observed between YouTube and TikTok videos according to the search terms, with respect to Global Quality Score (GQS), DISCERN, view counts, likes, comments, video duration, and subscriber numbers (*p* < 0.05).

When comparing platforms for the ‘Orthognathic Surgery’ term, TikTok videos showed slightly higher mean GQS and DISCERN scores compared to YouTube, though these differences were not statistically significant (p > 0.05). YouTube videos were longer in duration for certain comparisons. However, for the ‘Jaw Surgery’ term, YouTube demonstrated significantly longer video duration (*p* < 0.05), while TikTok videos demonstrated significantly greater engagement metrics including views, likes, comments, and subscriber numbers (*p* < 0.05).

The DISCERN instrument used here evaluates the reliability of health information based on citation of sources, balance, and evidence presentation; thus, higher DISCERN scores indicate more trustworthy and verifiable content.

These findings align with Table 3, which shows that platform differences varied by search term and metric type. While some comparisons showed statistical significance (e.g., duration and engagement metrics for ‘Jaw Surgery’), others did not reach significance (e.g., GQS and DISCERN scores across platforms for ‘Orthognathic Surgery’). [Table 3].

**Fig. 1:**
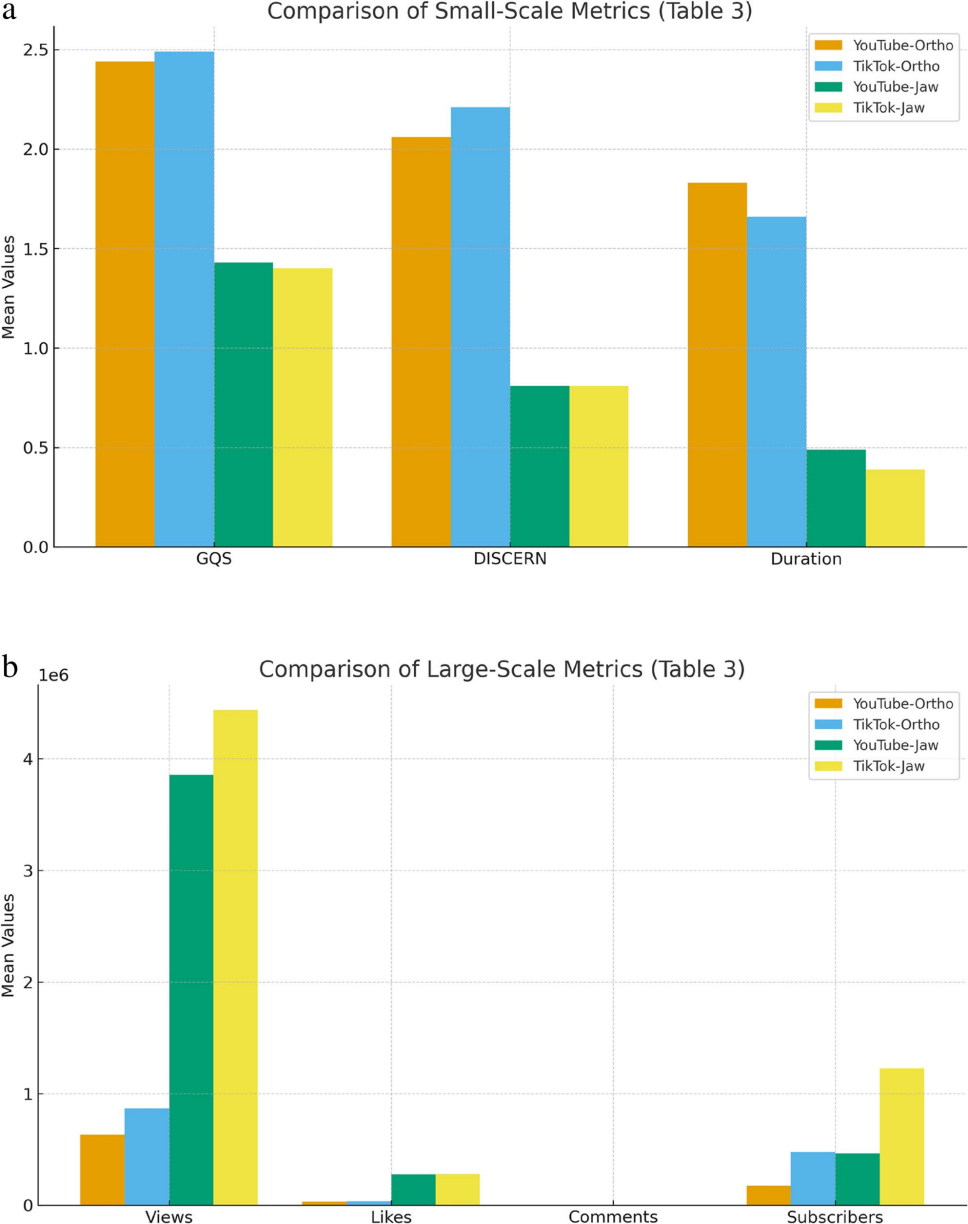
**a**. Comparison of small-scale metrics (GQS, DISCERN, Duration) across platforms and search terms. **b**. Comparison of large-scale metrics (Views, Likes, Comments, Subscribers) across platforms and search terms

Non-significant comparisons are reported to provide full transparency regarding cross-platform effects. Figure 1.

**Table 3:** Distribution and comparison of video characteristics (GQS, DISCERN, views, likes, comments, duration, and subscribers) according to social media platform and search terms (p-values are shown; values < 0.05 considered statistically significant)

		Orthognathic Surgery		Jaw Surgery		Between the terms	
		Min.-Max	Mean.±S.D.(Median)	Min.-Max	Mean.±S.D.(Median)	Test Statistics	*p*
YouTube	GQS	1–4	2.44 ± 0.91(3)	1–3	1.43 ± 0.63(1)	1327.0	< 0.001*
	DISCERN	0–4	2.06 ± 1.2(2)	0–3	0.81 ± 0.87(1)	1398.5	< 0.001*
	Views	261-32000000	631053.2 ± 3841450.43(22000)	1489-47700000	3854850.99 ± 8016402.23(442350)	933.0	< 0.001*
	Likes	0-2100000	31914.5 ± 238823.85(136)	39-5200000	279040.46 ± 759114.56(5724.5)	626.0	< 0.001*
	Comments	0-7650	219.28 ± 1057.54(19.5)	0-128800	2682.46 ± 14463.98(115.5)	1695.0	< 0.001*
	Duration	0.33–3.93	1.83 ± 0.91(1.87)	0.1–1.95	0.49 ± 0.3(0.43)	450.5	< 0.001*
	Number of subscribers	82-5130000	173829.78 ± 689811.71(14200)	36-14700000	466633.7 ± 1729709.33(91500)	1963.0	< 0.001*
TikTok	GQS	1–4	2.49 ± 0.99(3)	1–4	1.4 ± 0.7(1)	1302.0	< 0.001*
	DISCERN	0–4	2.21 ± 1.19(2)	0–3	0.81 ± 0.94(1)	1245.5	< 0.001*
	Views	804-18000000	870089.93 ± 2976209.03(21000)	1912-60900000	4438266.98 ± 9661052.73(513850)	1320.5	< 0.001*
	Likes	2-798000	35298.03 ± 132397.33(145)	39-5200000	280740.66 ± 816954.07(8720)	1037.5	< 0.001*
	Comments	0-7660	342.85 ± 1194.63(21.5)	0-128800	2405.54 ± 14373.76(120)	1813.0	< 0.001*
	Duration	0.33–3.97	1.66 ± 1.03(1.68)	0.07-3	0.39 ± 0.4(0.28)	468.0	< 0.001*
	Number of subscribers	154-6410000	476385.41 ± 1409001.55(14200)	36-14700000	1229773.85 ± 3897737.23(11600)	2899.0	< 0.001*
Inter-social media		Test Statistics	p	Test Statistics	p		
	GQS	3136.0	0.817	3046.0	0.521		
	DISCERN	3035.0	0.554	3142.5	0.841		
	Views	2837.5	0.216	3147.5	0.858		
	Likes	2675.5	0.073	3114.0	0.769		
	Comments	2706.0	0.092	2996.0	0.486		
	Duration	2556.5	0.177	2275.0	0.002*		
	Number of subscribers	2605.0	0.038*	2279.5	0.002*		

*p < 0.05

### Search term-based comparisons

**Fig. 2:**
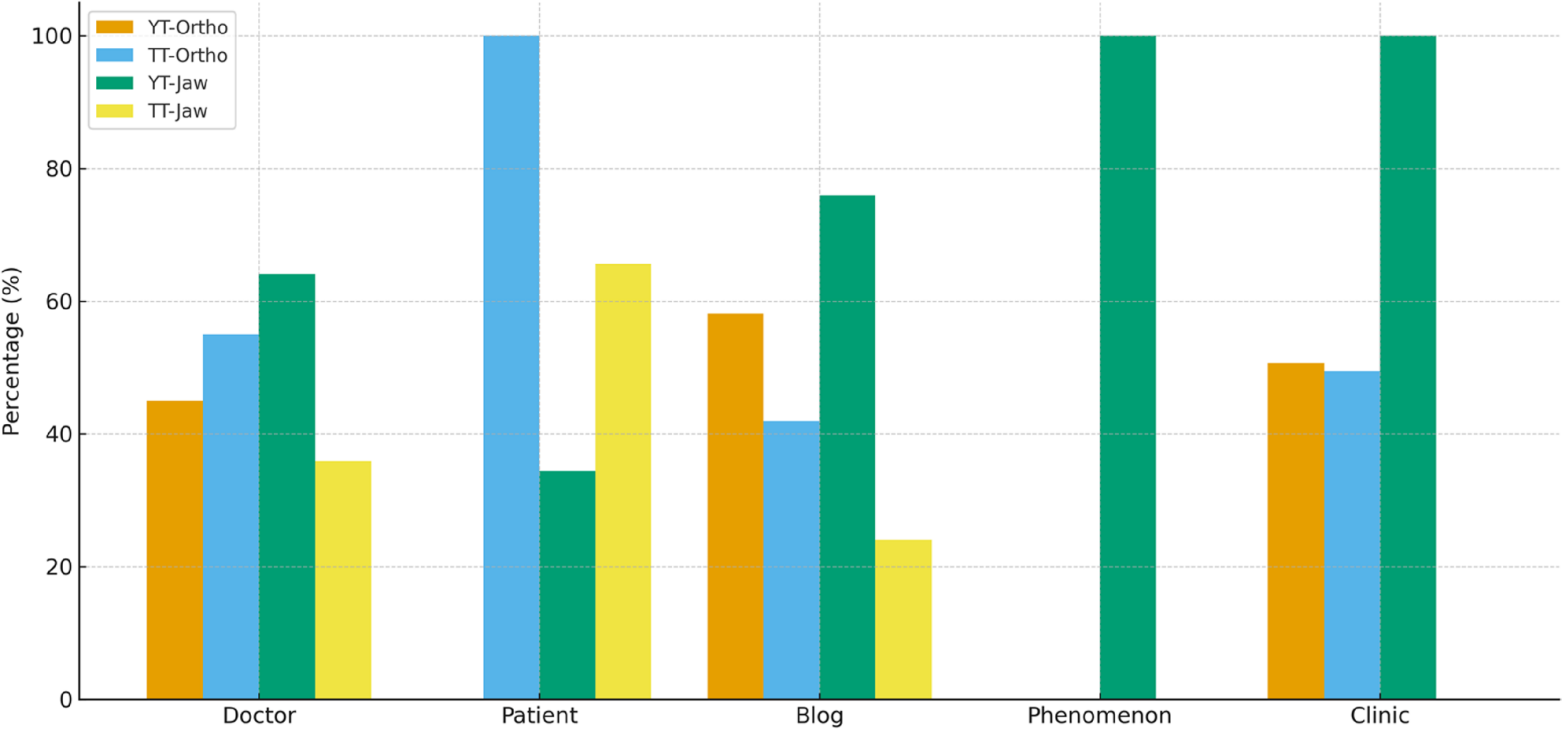
Distribution of video uploader types across YouTube and TikTok for the search terms *“Orthognathic Surgery”* and *“Jaw Surgery.”* The bars represent the percentage of videos uploaded by doctors, patients, blogs, content creators/influencers, and clinics, highlighting platform- and term-specific differences

Statistically significant associations were identified between the Jaw Surgery search term, uploader type, and platform (*p* < 0.05). TikTok was predominantly used by patients, whereas YouTube was more commonly utilized by doctors and bloggers. No significant association was observed between uploader type and platform for the Orthognathic Surgery term (*p* > 0.05). As shown in Figure 2, the distribution of uploader types differed significantly between platforms and search terms.

For both platforms, search terms and uploader categories showed significant associations (*p* < 0.05):

- On YouTube, all clinical uploaders used the Orthognathic Surgery term, while patients exclusively used the Jaw Surgery term.
- On TikTok, clinical and blog uploaders primarily used the Orthognathic Surgery term, whereas patients predominantly used the Jaw Surgery term [Table 4].

**Table 4:** Relationships between social media type. search terms and distribution of video uploader

Search Term		YouTube			TikTok					
	Uploader	n	%	%S.M.	n	%	%S.M.	Test Statistics	*p*	
Orthognathic Surgery		Case Evaluation	30	52.6	37.5	27	47.4	33.8	10.820	0.029*
		Animation	18	42.9	22.5	24	57.1	30.0		
		Experience	29	61.7	36.3	18	38.3	22.5		
		Surgery	1	100.0	1.3	0	0.0	0.0		
		Change	2	15.4	2.5	11	84.6	13.8		
Jaw Surgery		Case Evaluation	31	100.0	38.8	0	0.0	0.0	57.254	< 0.001*
		Animation	15	48.4	18.8	16	51.6	20.0		
		Experience	0	0.0	0.0	24	100.0	30.0		
		Change	34	47.2	42.5	38	52.8	47.5		
		Recommendation	0	0.0	0.0	2	100.0	2.5		
Between the terms		Test Statistics	70.600			53.137				
		p	< 0.001*			< 0.001*				
Total		Case Evaluation	61	69.3	38.1	27	30.7	16.9	20.906**	< 0.001*
		Animation	33	45.2	20.6	40	54.8	25.0		
		Experience	29	40.8	18.1	42	59.2	26.3		
		Surgery	1	100.0	0.6	0	0.0	0.0		
		Change	36	42.4	22.5	49	57.6	30.6		
		Recommendation	0	0.0	0.0	2	100.0	1.3		

*p < 0.05. **: Fisher’s Exact test. %: Row percentage and %S.M.: Column percentage for social media

This finding indicates a distinction between professional vs. experiential content depending on both the platform and keyword used.

### Uploader and content analysis

**Fig. 3:**
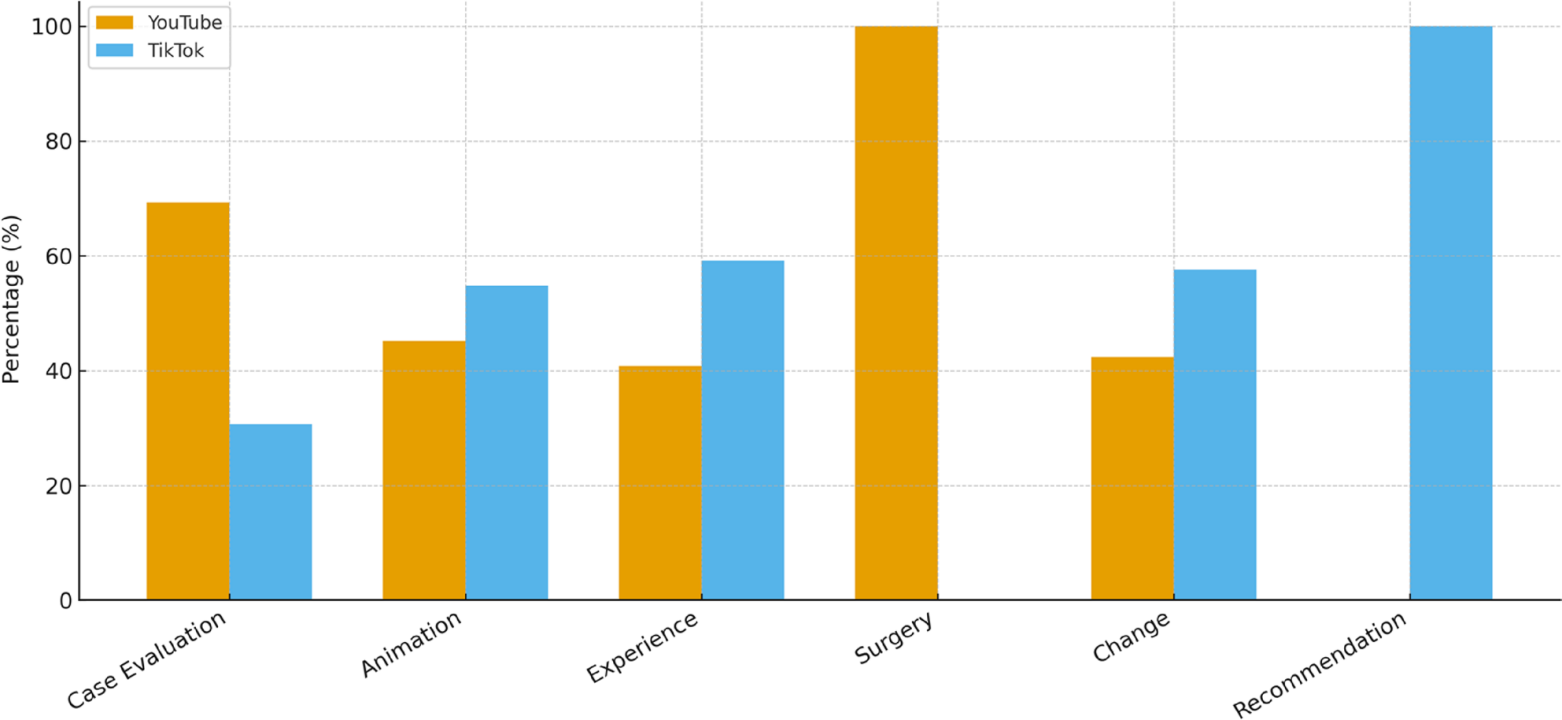
Distribution of video content types across YouTube and TikTok. The bars represent the percentage of videos categorized as case evaluation, animation, experience, surgery, change, and recommendation, demonstrating platform-specific differences in content focus

Statistically significant relationships were identified between the Orthognathic Surgery and Jaw Surgery search terms, the content type in all videos, and the social media platforms (*p* < 0.05). As shown in Figure 3, the distribution of content types differed significantly across platforms and search terms, with YouTube favoring case evaluations and TikTok focusing more on experience- and transformation-based content.

TikTok™ was mainly used for transformation-focused content under the Orthognathic Surgery term, while YouTube™ was predominantly used for experience-based and case evaluation content. For the Jaw Surgery term, TikTok™ was used primarily for case experiences, while YouTube™ was utilized mainly for case evaluations. These differences were statistically significant (*p* < 0.05) [Table 5].

**Table 5:** Distribution of video content and relationships between social media type and search terms

		YouTube			TikTok				
Search Term	Uploader	n	%	%S.M.	n	%	%S.M.	Test Statistics	p
Orthognathic Surgery	Case Evaluation	30	52.6	37.5	27	47.4	33.8	10.820	0.029*
	Animation	18	42.9	22.5	24	57.1	30.0		
	Experience	29	61.7	36.3	18	38.3	22.5		
	Surgery	1	100.0	1.3	0	0.0	0.0		
	Change	2	15.4	2.5	11	84.6	13.8		
Jaw Surgery	Case Evaluation	31	100.0	38.8	0	0.0	0.0	57.254	<0.001*
	Animation	15	48.4	18.8	16	51.6	20.0		
	Experience	0	0.0	0.0	24	100.0	30.0		
	Change	34	47.2	42.5	38	52.8	47.5		
	Recommendation	0	0.0	0.0	2	100.0	2.5		
Between the terms	Test Statistics	70.600			53.137				
	p	<0.001*			<0.001*				
Total	Case Evaluation	61	69.3	38.1	27	30.7	16.9	20.906**	<0.001*
	Animation	33	45.2	20.6	40	54.8	25.0		
	Experience	29	40.8	18.1	42	59.2	26.3		
	Surgery	1	100.0	0.6	0	0.0	0.0		
	Change	36	42.4	22.5	49	57.6	30.6		
	Recommendation	0	0.0	0.0	2	100.0	1.3		

*p<0.05. **: Fisher’s Exact test. %: Row percentage and %S.M.: Column percentage for social media

In both platforms, the thematic distinction was consistent: TikTok™ emphasized short, subjective “before-and-after” transformations, whereas YouTube™ favored structured, informative case analyses. This underlines that content orientation varies not only by platform but also by terminology used.

In the videos retrieved with the “Orthognathic Surgery” search term, the GQS and DISCERN scores, as well as the average video duration, were higher than those retrieved with the “Jaw Surgery” term. Conversely, the “Jaw Surgery” term received higher numbers of views, likes, comments, and subscribers.

Although GQS and DISCERN were used to assess general quality and reliability, an additional content-specific framework was applied to determine whether the videos covered key topics relevant to orthognathic surgery (e.g., indications, surgical process, risks, recovery, and patient experience). Videos that addressed at least three of these core elements were considered to include important content.

Overall, videos retrieved with the “Orthognathic Surgery” search term exhibited higher GQS and DISCERN scores and longer durations, reflecting greater informational quality and reliability. Conversely, “Jaw Surgery” videos achieved higher engagement metrics (views, likes, comments, subscribers), indicating greater popularity but lower educational value. These findings suggest that content depth and engagement appeal are inversely related across the two search terms and platforms.

All videos were independently evaluated by two researchers: one orthodontic specialist with 8 years of clinical experience and one final-year dental student trained in the use of the scoring criteria. Disagreements in scoring were resolved by consensus through joint discussion.

In particular, TikTok videos were predominantly uploaded by patients and focused mainly on transformation and personal experience content, whereas YouTube videos were more frequently uploaded by clinicians and clinics, with a higher proportion of case evaluations. These differences highlight how the two platforms serve distinct roles: TikTok reflecting patient journeys and subjective perspectives, while YouTube providing more professional and evaluative content.

## Discussion

This study aimed to evaluate and compare the informational quality and educational value of orthognathic surgery-related content available on YouTube and TikTok. It provides a direct cross-platform comparison using standardized scoring tools such as GQS and a modified DISCERN index. The term “reliability” in this context reflects relative trustworthiness as inferred through content presentation and structure, rather than verification against clinical guidelines or expert consensus [22]. As DISCERN criteria emphasize aspects such as source citation, balance, and reference to evidence, low DISCERN values in both platforms suggest that most videos lacked transparent sourcing and balanced information presentation.

This study has several strengths. First, it represents one of the first direct comparative analyses of orthognathic surgery content across TikTok™ and YouTube™, addressing a gap in the literature. Second, the use of validated assessment tools (GQS and modified DISCERN) with excellent inter-rater reliability (Cohen’s kappa = 0.95) ensures standardized and reproducible evaluation. Third, the methodological rigor including incognito searches, search repetition for consistency verification, and transparent reporting of both significant and non-significant findings—enhances the credibility of our results. Finally, the sample size of 320 videos across two platforms and two search terms provides sufficient statistical power to detect meaningful differences in content quality and engagement patterns.

When the quality and informativeness of the videos were evaluated through the *Global Quality Score (GQS)* and *DISCERN* scores, it was found that videos labeled “Orthognathic Surgery” were of higher quality and more informative than videos labeled “Jaw Surgery” on social media platforms. This study suggests that videos specifically about orthognathic surgery offer higher quality and more informative content. Nevertheless, some comparisons between the two platforms and search terms did not yield statistically significant differences, indicating that both platforms share similar limitations regarding the depth and reliability of medical information. In the study by Szeto et al. [23], it was stated that YouTube videos are effective in providing health information. Although the findings suggest that videos retrieved with the ‘Orthognathic Surgery’ term exhibited higher GQS and DISCERN scores regardless of platform, overall quality remained poor to moderate on both YouTube and TikTok, with no consistent platform-based superiority in content quality. This broader inclusion better reflects the diversity of informational content across the two platforms. However, this pattern was not statistically significant for all search terms (*p* > 0.05 for ‘Orthognathic Surgery’). Features such as the number of views, likes, comments, and subscribers provide information about the popularity and interaction of the videos. The study shows that videos tagged with “Jaw Surgery” have higher views, likes, comments, and subscribers on both platforms. This result suggests that users search for and interact more with the search term “Jaw Surgery.” Literature indicates that social media users are generally more inclined to engage with more visual and concise information regarding health issues [24]. Short-video platforms such as TikTok appear to be well-suited for this type of content. TikTok’s demographic and rapid content cycle contribute to its broad reach. In a study by Lupton [25], the effects of social media platforms on user interaction were examined, with the conclusion that short-video platforms like TikTok engage users more. The higher scores for videos labeled “Orthognathic Surgery” indicate that these videos are better structured and richer in terms of information. The study by Covolo et al. [26] emphasizes that YouTube is an effective tool in health communication. This finding suggests that YouTube may provide relatively more informative and structured content.

Analysis of video duration revealed that videos tagged with “Orthognathic Surgery” were longer than those tagged with “Jaw Surgery” on both platforms. This result supports the notion that orthognathic surgery, which involves more complex and detailed information, requires longer videos. Previous studies have suggested that longer videos generally provide more detailed information and may be more effective for educational purposes [27]. However, longer duration did not necessarily correspond to higher engagement, supporting the hypothesis that informational depth and popularity are inversely related in social media health communication.

Regarding the uploader type and content distribution, significant differences were found between uploaders and content types on YouTube and TikTok platforms. Elmore et al. [28] reported that YouTube was more preferred by healthcare professionals. The results of this study also support this observation. Videos uploaded by clinics and physicians were more common on YouTube, whereas patients and bloggers uploaded more videos on TikTok. Additionally, case evaluation and experience-themed content labeled with “Orthognathic Surgery” was more prevalent, whereas transformation content was more common in videos labeled with “Jaw Surgery.” These results suggest that different social media platforms are preferred by distinct user groups and content. Previous studies have emphasized in the literature that YouTube is generally used for more professional and informative purposes, while TikTok is used for more personal and entertaining content [11]. The fact that videos under the “Orthognathic Surgery” tag focus on case evaluation and experience-based content shows that these videos are more informative and educational. This type of content may support viewers in understanding surgical and treatment processes. On the other hand, the focus of videos under the “Jaw Surgery” tag on transformation and case experiences suggests that these videos aim to inspire viewers and share personal experiences. These findings align with the conclusions drawn by Smith et al. [29] regarding the effects of social media content on users. Clinically, this distinction implies that while TikTok may serve as a motivational and experiential resource for patients, YouTube remains a more suitable platform for educational dissemination and pre-surgical counseling materials.

This study has limitations. First, only two social media platforms (YouTube and TikTok) were examined. While other platforms such as Instagram or Facebook also host video content, YouTube and TikTok were selected because of their high usage rates, algorithm-based content distribution, and primary focus on video format, making them the most relevant for comparison in this context. Including additional platforms would have introduced significant heterogeneity in content structure and engagement metrics, potentially compromising comparability. Second, social media algorithms can influence which videos are more visible to users, often favoring content with higher engagement metrics rather than educational quality. Third, the information provided in the videos was not cross-verified with clinical guidelines or expert opinion, which may affect the validity of the educational claims made in the content. Additionally, only English-language videos were included, which limits the generalizability of the findings to non-English-speaking populations. Furthermore, future research should incorporate multilingual datasets and real-time algorithmic tracking to better understand the evolving visibility of medically oriented content.

Other limitations of this study include the restricted range of keywords used during the data collection phase, the analysis being limited to videos published within a specific time frame, the inclusion criteria focused solely on content relevance and language, without restrictions on video length. Although video duration varied between platforms due to their inherent design, no artificial limit was imposed. This approach aimed to better capture the range of content available on each platform. While this approach allowed for better comparison with TikTok’s naturally short video format, it may have resulted in the exclusion of longer, potentially higher-quality content. The restriction was implemented to standardize video length across platforms and ensure methodological consistency; however, it may have affected the generalizability of the findings. Data analysis was performed independently by two researchers. Clinical trial number: not applicable. All videos were assessed on the same mobile device under consistent environmental conditions; however, the inability to blind the evaluators to the source platform due to visual and stylistic differences between TikTok and YouTube may have introduced bias.

Moreover, limiting the search terms to “orthognathic surgery” and “jaw surgery” may have excluded other relevant content. However, these terms were deliberately chosen because they are the most commonly used by both healthcare professionals and the general public, ensuring a focus on highly representative and frequently searched content. This methodological focus strengthens comparability but may not capture niche or specialized surgical topics often discussed in professional forums.

Furthermore, the cross-sectional nature of this study means that the findings represent a snapshot of available content during the data collection period (March-April 2024) and may not reflect subsequent changes in platform algorithms or content availability. The moderate inter-platform agreement in some metrics (*p* > 0.05) suggests that content quality issues may be systemic across social media rather than platform-specific. Finally, while GQS and DISCERN provide standardized quality assessment, they may not fully capture the subjective patient experience or the motivational value that transformation-focused content provides, which could be clinically relevant for patient engagement even if not educationally comprehensive.

Future studies can address these limitations by including a larger dataset and different platforms. Healthcare professionals and organizations should be more proactive in disseminating accurate and reliable health information on social media platforms. Informative content and the sharing of patient experiences can increase public awareness about health issues. Social media platforms should implement stricter fact-checking mechanisms to improve the accuracy and reliability of health-related videos. This will help protect users from misleading information. Platforms should also be more transparent about how their algorithms work and guide users towards accurate health information. Users should be informed about how to evaluate the credibility of health-related content on social media. Overall, the findings indicate a potential need for structured, evidence-based video production guidelines to bridge the gap between engagement-oriented and educational content in orthognathic surgery communication.

## Conclusion

This study comparatively evaluated the quality, reliability, and engagement metrics of orthognathic surgery-related videos on TikTok™ and YouTube™. The findings indicate that while quality and reliability scores varied by search term rather than by platform, TikTok™ videos tended to achieve greater user engagement, with significantly higher subscriber counts. However, content on both platforms was generally of poor to moderate quality with limited reliability, regardless of the search term or content source. Although both platforms provide easily accessible visual content about orthognathic surgery, many videos lacked scientific accuracy, clear referencing, and balanced information.

These findings indicate that social media users may be exposed to misleading or incomplete medical information when searching for surgical topics online.

Clinically, this highlights the need for healthcare professionals and academic institutions to actively participate in producing accurate, evidence-based educational content on video-sharing platforms.

Encouraging collaboration between clinicians and digital media creators could improve both accessibility and reliability of surgical information presented to the public.

In the future, platform-specific educational strategies (particularly on short-video applications) should be developed to promote accurate medical communication and enhance patient understanding before and after orthognathic surgery.

## Data Availability

The datasets generated and analyzed during the current study consist solely of publicly accessible online videos from YouTube™ and TikTok™. These materials are available from the corresponding author upon reasonable request.

## Abbreviations

GQS: Global Quality Score
IRB: Institutional Review Board
VPN: Virtual Private Network
SPSS: Statistical Package for the Social Sciences
DISCERN: a validated instrument for evaluating the quality of health information
